# Siglec-15 Expression in Gastric Carcinoma and Its Association With Clinicopathological Variables

**DOI:** 10.14740/wjon2749

**Published:** 2026-09-04

**Authors:** Sara Al Badr, Abdelkader Battah, Ahmad Alsughayer, Fanar Alsmarat, Khalid Halahleh, Maher Sughayer

**Affiliations:** a Department of Pathology and Laboratory Medicine, King Hussein Cancer Center, Amman 11941, Jordan; b School of Medicine, University of Jordan, Amman 11941, Jordan; c Department of Surgery, King Hussein Cancer Center, Amman 11941, Jordan; d Department of Anatomy, Physiology and Biochemistry, Faculty of Medicine, The Hashemite University, Zarqa 13133, Jordan; e Department of Medicine, King Hussein Cancer Center, Amman, Jordan

**Keywords:** Siglec-15, Gastric carcinoma, Immune checkpoint

## Abstract

**Background:**

Gastric cancer remains the fifth most common malignancy worldwide and is often diagnosed at advanced stages with limited treatment options. Although immunotherapy targeting the programmed cell death protein-1 (PD-1)/programmed death-ligand 1 (PD-L1) has improved outcomes in several cancers, many patients exhibit resistance, necessitating alternative therapeutic strategies. Sialic acid-binding immunoglobulin-like lectin-15 (Siglec-15), a recently recognized immune suppressor, is overexpressed in tumor cells and tumor-associated macrophages (TAMs), and is largely absent from normal tissues, making it a promising immunotherapy target.

**Methods:**

This tissue microarray (TMA) study retrospectively collected formalin-fixed paraffin-embedded (FFPE) tissue specimens from 2015 to 2019 for 77 gastric cancer patients from King Hussein Cancer Center. Siglec-15 expression patterns in tumor cells and TAMs were analyzed and compared with clinicopathological features and patient outcomes. Survival was assessed using Kaplan–Meier analysis.

**Results:**

Siglec-15 expression was detected in 50.7% of tumor samples and 36.4% of TAMs. Membranous expression was significantly associated with tumor differentiation, with higher expression observed in moderately differentiated tumors (P = 0.048), while cytoplasmic and TAM expression showed no significant associations with clinical variables. Although not statistically significant, positive Siglec-15 expression—both membranous and cytoplasmic—was associated with shorter median overall survival (cytoplasmic: 30.62 vs. 74.92 months, P = 0.1790; membranous: 39.44 vs. 66.07 months, P = 0.5761). Established prognostic indicators such as age, clinical stage, and pT stage were significantly associated with overall survival.

**Conclusion:**

Siglec-15 is frequently expressed in gastric cancer and may contribute to immune evasion within the tumor microenvironment. Although no significant association with prognosis was found, its high expression underscores its potential as a novel immunotherapeutic target. Further studies are warranted to validate these findings and explore Siglec-15 clinical utility.

## Introduction

Gastric cancer ranks as the fifth most common cancer globally in both incidence and mortality [1]. The majority of gastric cancer patients are diagnosed at advanced stages, when treatment options are limited and the prognosis is poor [2].

Recent advances in cancer immunotherapy, particularly the blockade of programmed cell death protein-1 (PD-1) and its ligand PD-L1, have revolutionized treatment outcomes for cancer patients by reestablishing anti-tumor immune responses within the tumor microenvironment (TME) [3]. However, many patients exhibit either primary resistance or eventually develop acquired resistance to PD-1/PD-L1 blockades [4]. Moreover, this pathway addresses only a subset of mechanisms underlying tumor-induced immune evasion, underscoring the need for alternative or complementary immunotherapeutic targets [4].

The sialic acid-binding immunoglobulin-like lectin-15 (Siglec-15) is a transmembrane protein primarily expressed on macrophages, dendritic cells, and osteoclasts [5]. Notably, compared to normal tissues, Siglec-15 is markedly overexpressed in cancer cells and tumor-associated macrophages (TAMs) [6]. Within TME, Siglec-15 suppresses cytotoxic T-cell activation and proliferation, aiding immune evasion [7]. It also promotes immune suppression via transforming growth factor-β (TGF-β) secretion through the DAP12–Syk pathway [8]. Siglec-15 and PD-L1 expression have been reported to be mutually exclusive, suggesting that Siglec-15 may serve as a promising immunotherapeutic target in PD-L1-negative tumors or in patients who are resistant to PD-1/PD-L1 blockade [8].

Siglec-15 expression has been observed in several cancers, including breast cancer, pancreatic adenocarcinoma, head and neck squamous cell carcinoma, and uterine carcinosarcoma [9]. However, current data on its expression frequency in gastric cancer, its prognostic value, and its association with clinicopathological features are limited and controversial [10]. Therefore, this study aimed to investigate Siglec-15 expression in gastric cancer and examine its association with clinicopathological features and patient prognosis.

## Materials and Methods

### Patients and samples

Following approval from the Institutional Review Board (IRB), formalin-fixed paraffin-embedded (FFPE) tissue blocks were obtained from patients who underwent partial or total gastrectomy at King Hussein Cancer Center (KHCC) between 2010 and 2018. A total of 100 cases of 125 available cases were randomly selected for initial review. For each selected case, corresponding pathology reports, FFPE tumor blocks, and hematoxylin and eosin (H&E)-stained sections were thoroughly examined. Additional clinical data, including overall survival, clinical stage, and smoking history, were extracted from patients’ medical records.

Upon preliminary review, patients diagnosed with non-gastric cancers or with gastric tumors other than adenocarcinomas were removed (n = 8). To preserve the integrity of tissue samples during tissue microarray (TMA) construction, blocks with significant fragmentation were also excluded (n = 7). The remaining 85 cases were examined by one of the researchers (MAS) using several H&E slides. The precise tissue region of interest was determined, and the block with the greatest cancer cell count was selected for TMA production after the examination. Cases with a low number of cancer cells on all available slides were also eliminated to decrease the risk of false-negative results (n = 8). The remaining 77 cases were used to make the TMA blocks. All relevant clinicopathological and histological data were documented in an organized Excel spreadsheet for subsequent analyses.

### TMA

Four TMA blocks were constructed and utilized in this study, each containing between 20 and 30 tissue cores, along with at least one control core derived from tonsillar tissue. From each TMA block, two 4 µm sections were obtained. The first section was stained with H&E to assess the adequacy and integrity of the tumor cores. The second section, intended for immunohistochemistry (IHC), was made on positively charged slides.

Duplicate cores from the same case were included in instances where tissue quality was suboptimal or the tumor location within the core was uncertain. If a core was lost during sectioning, a replacement core from the corresponding original FFPE block was extracted and incorporated into the final TMA, ensuring near-complete representation of the initially selected cases.

### IHC procedure and evaluation

According to the manufacturer’s recommendations, TMA slides were immunostained using an anti-Siglec-15 antibody (rabbit polyclonal antibody PA5-116878, Thermo Fisher Scientific, USA) on the Ventana Benchmark Ultra platform (Roche, Tucson, AZ). Both positive and negative controls were included on every slide to ensure staining reliability.

The IHC evaluation was performed by one of the researchers (MAS) using a manual precise count method by examining the cores at × 200 magnification and counting no fewer than 100 cancer cells and TAM cells in each case using a light microscope. Siglec-15-positive immunolabeling was predominantly observed as membranous and/or cytoplasmic staining in tumor cells, while TAM exhibited linear membranous, diffuse, and/or punctate cytoplasmic staining. The cut-off values used in the study are as follows: 40% for cytoplasmic staining, 20% for membranous staining, and 20% for TAM staining. A positive cell for TAM and cytoplasmic staining includes any intensity and for membranous staining any intensity and either partial or complete membranous staining.

### Statistical analyses

Descriptive statistics were employed to summarize the patients’ demographic and clinical characteristics. Comparisons between Siglec-15 positive and negative cases with respect to various risk factors were conducted using Fisher’s exact test. For the survival analysis, Kaplan–Meier curves were generated, with a significance level set at P ≤ 0.05. All statistical analyses were performed using SAS version 9.4 (SAS Institute Inc., Cary, NC).

### IRB approval

The IRB at KHCC has approved this study under the Reference No. 24 KHCC 130. The study was conducted in compliance with the ethical standards of the responsible institution on human subjects as well as with the Helsinki Declaration.

## Results

### Patient characteristics and expression of Siglec-15

The study included predominantly male patients (n = 48, 62.3%). The median age for the study cohort was 65 years and the interquartile was 25. At the time of analysis, 44 patients (57.1%) were deceased. According to Laurén’s classification, the diffuse and indeterminate subtypes were the most prevalent (n = 58, 75.3%), while the intestinal subtype accounted for 19 cases (24.7%). The majority of tumors (54.5%) were poorly differentiated, while 42.9% were moderately differentiated. Among 61 patients with documented clinical stages, 40 cases (65.6%) were diagnosed at stage III or higher. Gastritis was present in 13 cases (16.9%) (Table 1).

**Table 1:** Demographic, Clinicopathological, and Molecular Characteristics of Gastric Cancer Patients

Variable	Number	Percentage (%)
Gender		
Female	29	37.7%
Male	48	62.3%
Differentiation		
NA	2	
Moderate	33	44.0%
Poor	42	56.0%
Clinical stage		
NA	16	
I	6	9.8%
II	15	24.6%
III	31	50.8%
IV	9	14.8%
Sigent cell		
NA	1	
No	53	69.7%
Yes	23	30.3%
Location		
NA	3	
Distal	38	51.3%
Proximal	23	31.1%
Whole	13	17.6%
Neoadjuvant therapy		
No	51	66.2%
Yes	26	33.8%
Gastritis		
No	64	83.1%
Yes	13	16.9%
*H. pylori*		
No	69	89.6%
Yes	8	10.4%
Lymphovascular invasion		
Yes	40	51.9%
No	37	48.1%
PD-L1		
NEG	26	33.8%
POS	51	66.2%
pT		
NA	1	
T1	6	7.9%
T2	13	17.1%
T3	28	36.8%
T4	29	38.2%
pN		
NA		
N positive	57	76%
N0	18	24.0%
pM		
NA	1	
0	44	57.9%
1	32	42.1%
Laurén classification		
Diffuse	29	37.7%
Indeterminate	29	37.7%
Intestinal	19	24.6%
Cytoplasmic Siglec-15 expression in GC cells		
Negative	44	57.1%
Positive (≥ 40%)	33	42.9%
Membranous Siglec-15 expression in GC cells		
Negative	51	66.2%
Positive (≥ 20%)	26	33.8%
Siglec-15 expression in TAM cells		
Negative	49	63.6%
Positive (≥ 20%)	28	36.4%

GC: gastric cancer; NA: not available; PD-L1: programmed death-ligand 1; TAM: tumor-associated macrophage.

**Figure 1:**
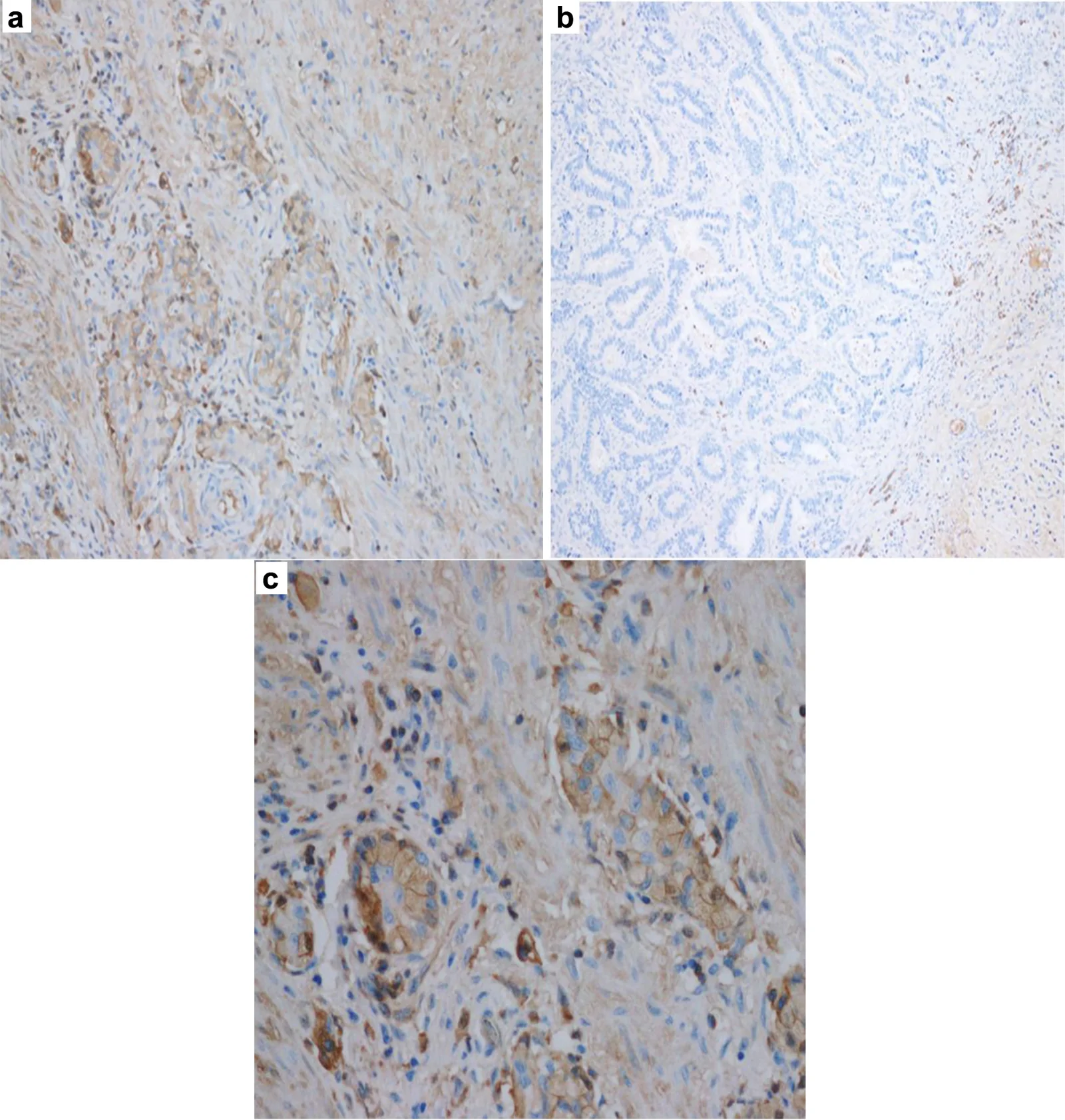
Representative immunohistochemistry images of Siglec-15 expression in gastric cancer tissues. (a) Positive Siglec-15 expression. (b) Negative Siglec-15 expression. (c) Positive membranous and cytoplasmic expression in tumor cells and Positive expression in tumor-associated macrophages (TAMs).

Siglec-15 expression was detected in the cytoplasm and/or membranes of gastric cancer cells in 39 patients (50.65%), including 14 females (18.18%) and 25 males (32.47%). Specifically, Siglec-15 was observed in the membranes of tumor cells in 26 patients (33.8%) and in the cytoplasm of 33 patients (42.9%). Additionally, Siglec-15 expression was detected in TAMs in 28 patients (36.4%) (Table 1, Fig. 1).

### Association of Siglec-15 expression with clinicopathological factors in gastric cancer

Membranous Siglec-15 expression in gastric cancer cells was significantly associated with tumor differentiation (P = 0.048), with higher expression observed in moderately differentiated tumors (Table 2). However, no significant associations were found with age, gender, clinical stage, signet ring cell histology, tumor location, neoadjuvant therapy, gastritis, *H. pylori* infection, lymphovascular invasion, PD-L1 status, pT, pN, pM, or Laurén classification. Cytoplasmic Siglec-15 expression did not demonstrate significant associations with clinicopathological factors, though a trend was observed toward higher expression in tumors with lymphovascular invasion (P = 0.075). Similarly, Siglec-15 expression in TAMs showed no significant association with any clinicopathological factors (Table 3).

**Table 2:** Membranous and Cytoplasmic Siglec-15 Expression and Its Association With Various Clinical Variables in Gastric Cancer Patients

Variable	Category	Membranous		P-value	Cytoplasmic		P-value
		Negative (%)	Positive (%)		Negative (%)	Positive (%)	
Age (years)	≤ 65	27 (52.9%)	14 (53.8%)	0.940	21 (47.7%)	20 (60.6%)	0.262
	> 65	24 (47.1%)	12 (46.2%)		23 (52.3%)	13 (39.4%)	
Gender	Female	20 (39.2%)	9 (34.6%)	0.694	16 (36.4%)	13 (39.4%)	0.786
	Male	31 (60.8%)	17 (65.4%)		28 (63.6%)	20 (60.6%)	
Differentiation	Moderate	18 (36.0%)	15 (60.0%)	0.048*	18 (42.9%)	15 (45.5%)	0.822
	Poor	32 (64.0%)	10 (40.0%)		24 (57.1%)	18 (54.5%)	
Clinical stage	I & II	12 (30.0%)	9 (42.9%)	0.315	12 (35.3%)	9 (33.3%)	0.873
	III & IV	28 (70.0%)	12 (57.1%)		22 (64.7%)	18 (66.7%)	
Signet ring cell	No	32 (64.0%)	21 (80.8%)	0.131	30 (69.8%)	23 (69.7%)	0.995
	Yes	18 (36.0%)	5 (19.2%)		13 (30.2%)	10 (30.3%)	
Location	Distal	26 (54.2%)	12 (46.2%)	0.616	25 (59.5%)	13 (40.6%)	0.199
	Proximal	13 (27.1%)	10 (38.5%)		12 (28.6%)	11 (34.4%)	
	Whole	9 (18.8%)	4 (15.4%)		5 (11.9%)	8 (25.0%)	
Neoadjuvant therapy	No	35 (68.6%)	16 (61.5%)	0.534	32 (72.7%)	19 (57.6%)	0.164
	Yes	16 (31.4%)	10 (38.5%)		12 (27.3%)	14 (42.4%)	
Gastritis	No	41 (80.4%)	23 (88.5%)	0.525	35 (79.5%)	29 (87.9%)	0.376
	Yes	10 (19.6%)	3 (11.5%)		9 (20.5%)	4 (12.1%)	
*H. pylori*	No	45 (88.2%)	24 (92.3%)	0.710	39 (88.6%)	30 (90.9%)	1.000
	Yes	6 (11.8%)	2 (7.7%)		5 (11.4%)	3 (9.1%)	
Lymphovascular invasion	Yes	24 (47.1%)	16 (61.5%)	0.229	19 (43.2%)	21 (63.6%)	0.075
	No	27 (52.9%)	10 (38.5%)		25 (56.8%)	12 (36.4%)	
PD-L1	Negative	20 (39.2%)	6 (23.1%)	0.157	14 (31.8%)	12 (36.4%)	0.676
	Positive	31 (60.8%)	20 (76.9%)		30 (68.2%)	21 (63.6%)	
pT stage	T1 & T2	14 (28.0%)	5 (19.2%)	0.402	14 (32.6%)	5 (15.2%)	0.111
	T3 & T4	36 (72.0%)	21 (80.8%)		29 (67.4%)	28 (84.8%)	
pN status	N0	12 (24.5%)	6 (23.1%)	0.892	12 (27.9%)	6 (18.8%)	0.358
	N+	37 (75.5%)	20 (76.9%)		31 (72.1%)	26 (81.3%)	
pM status	M0	26 (52.0%)	18 (69.2%)	0.149	21 (48.8%)	23 (69.7%)	0.068
	M1	24 (48.0%)	8 (30.8%)		22 (51.2%)	10 (30.3%)	
Laurén type	Diffuse	22 (43.1%)	7 (26.9%)	0.365	15 (34.1%)	14 (42.4%)	0.501
	Indeterminate	18 (35.3%)	11 (42.3%)		16 (36.4%)	13 (39.4%)	
	Intestinal	11 (21.6%)	8 (30.8%)		13 (29.5%)	6 (18.2%)	

*Statistically significant. PD-L1: programmed death-ligand 1.

**Table 3:** TAM Siglec-15 Expression and Its Association With Various Clinical Variables in Gastric Cancer Patients

Variable	Category	Negative (%)	Positive (%)	P-value
Age (years)	≤ 65	24 (49.0%)	17 (60.7%)	0.321
	> 65	25 (51.0%)	11 (39.3%)	
Gender	Female	18 (36.7%)	11 (39.3%)	0.824
	Male	31 (63.3%)	17 (60.7%)	
Differentiation	Moderate	20 (41.7%)	13 (48.1%)	0.587
	Poor	28 (58.3%)	14 (51.9%)	
Clinical stage	I & II	16 (41.0%)	5 (22.7%)	0.149
	III & IV	23 (59.0%)	17 (77.3%)	
Signet ring cell	No	32 (66.7%)	21 (75.0%)	0.446
	Yes	16 (33.3%)	7 (25.0%)	
Location	Distal	26 (56.5%)	12 (42.9%)	0.447
	Proximal	12 (26.1%)	11 (39.3%)	
	Whole	8 (17.4%)	5 (17.9%)	
Neoadjuvant therapy	No	32 (65.3%)	19 (67.9%)	0.820
	Yes	17 (34.7%)	9 (32.1%)	
Gastritis	No	42 (85.7%)	22 (78.6%)	0.421
	Yes	7 (14.3%)	6 (21.4%)	
*H. pylori*	No	44 (89.8%)	25 (89.3%)	1.000
	Yes	5 (10.2%)	3 (10.7%)	
Lymphovascular invasion	Yes	22 (44.9%)	18 (64.3%)	0.101
	No	27 (55.1%)	10 (35.7%)	
PD-L1	Negative	20 (40.8%)	6 (21.4%)	0.084
	Positive	29 (59.2%)	22 (78.6%)	
pT stage	T1 & T2	15 (31.3%)	4 (14.3%)	0.169
	T3 & T4	33 (68.8%)	24 (85.7%)	
pN status	N0	13 (27.1%)	5 (18.5%)	0.404
	N+	35 (72.9%)	22 (81.5%)	
pM status	M0	28 (58.3%)	16 (57.1%)	0.919
	M1	20 (41.7%)	12 (42.9%)	
Laurén type	Diffuse	20 (40.8%)	9 (32.1%)	0.720
	Indeterminate	18 (36.7%)	11 (39.3%)	
	Intestinal	11 (22.4%)	10 (28.6%)	

PD-L1: programmed death-ligand 1; TAM: tumor-associated macrophage.

### Siglec-15 expression and overall survival

**Figure 2:**
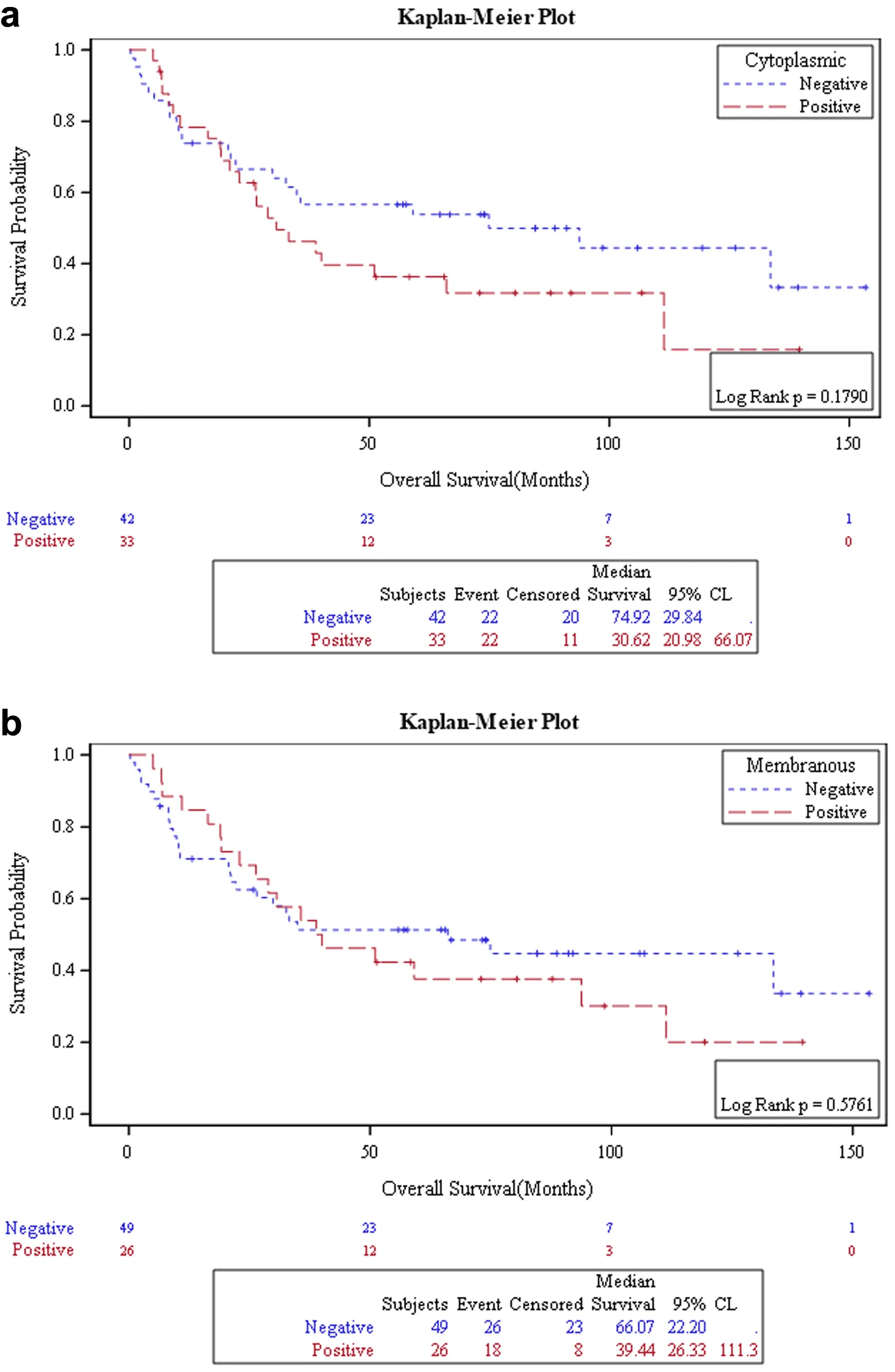
Kaplan–Meier curve analysis of overall survival in gastric cancer patients. (a) Association between cytoplasmic Siglec-15 expression and overall survival. (b) Association between membranous Siglec-15 expression and overall survival.

Patients with positive cytoplasmic or membranous Siglec-15 expression had shorter median overall survival, though the differences were not statistically significant (P = 0.1790 and P = 0.5761, Fig. 2). Median overall survival was 30.62 months for patients with positive cytoplasmic Siglec-15 expression compared to 74.92 months for patients with negative cytoplasmic Siglec-15 expression, while patients with positive Siglec-15 membranous expression had a median survival of 39.44 months compared to 66.07 months for patients with negative membranous Siglec-15 expression.

The 5-year survival rate was lower in patients with positive cytoplasmic Siglec-15 expression (36.3%, 95% confidence interval (CI): 20.5–53.7) compared to patients with negative cytoplasmic Siglec-15 expression (53.8%, 95% CI: 38.5–68.7). A similar trend was observed for membranous Siglec-15 expression, with 5-year survival rates of 37.6% (95% CI: 20.0–57.0) in positive membranous Siglec-15 expression patients versus 51.3% (95% CI: 37.1–65.4) in negative membranous Siglec-15 expression patients.

**Figure 3:**
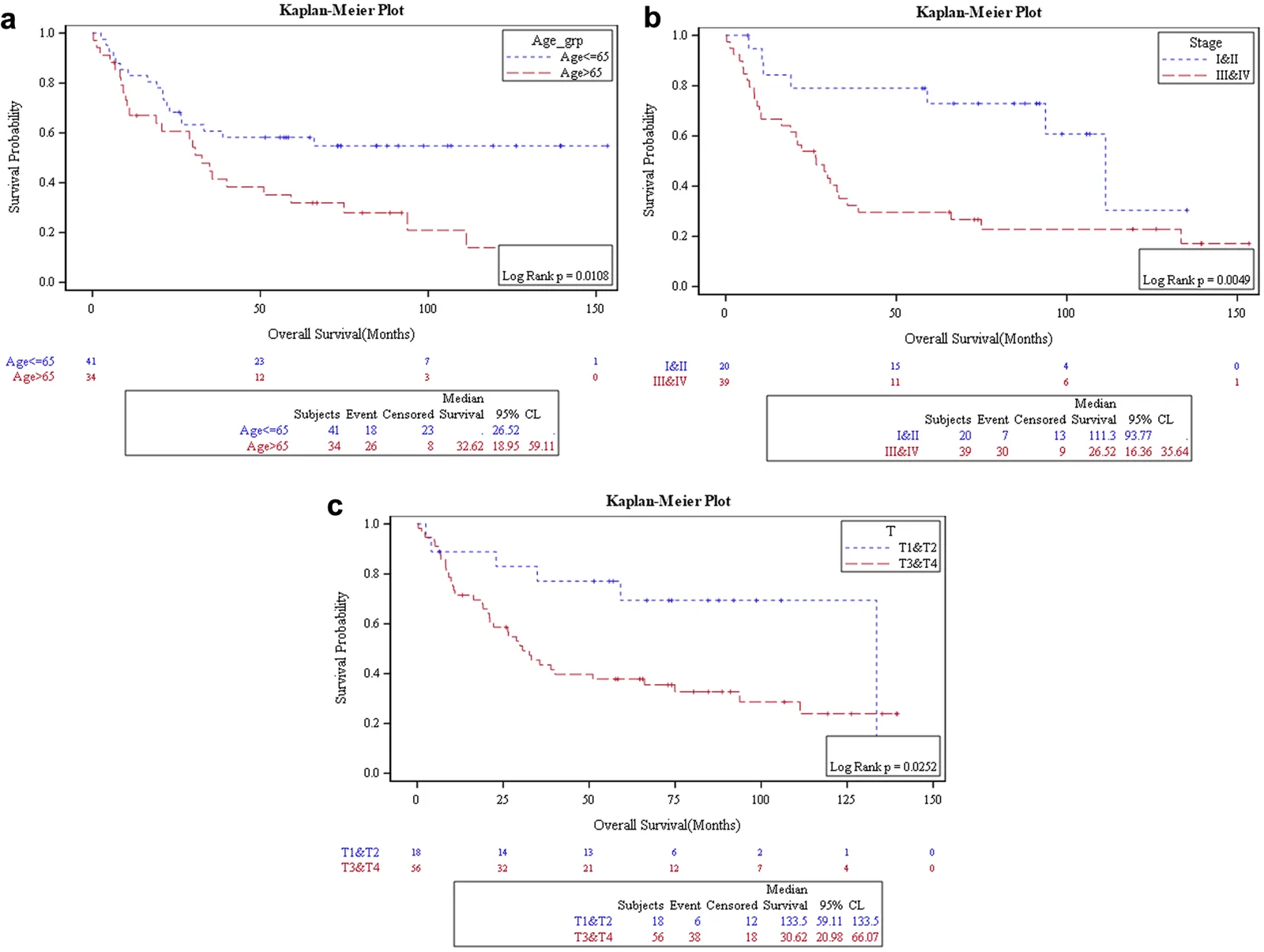
Kaplan–Meier curve analysis of overall survival in gastric cancer patients. (a) Association between age and overall survival. (b) Association between clinical stage and overall survival. (c) Association between pathological T stage and overall survival.

Several established clinicopathological factors were significantly associated with overall survival in gastric cancer patients. Advanced age was found to be significantly associated with poorer median overall survival (P = 0.0108, Fig. 3a). Additionally, patients diagnosed at early stages (I/II) experienced significantly longer median overall survival compared to patients diagnosed at advanced stages (III/IV) (P = 0.0049, Fig. 3b). Furthermore, a higher pT stage was significantly associated with decreased median overall survival (P = 0.0252, Fig. 3c).

## Discussion

Siglec-15 is a promising novel target for immunotherapy to restore anti-tumor immunity within the TME. Unlike other immune suppressors, Siglec-15 is poorly expressed in normal tissues but highly expressed in TAMs and cancer cells, making it a selective target for therapy [7, 11, 12]. Preclinical studies in mouse models have demonstrated that Siglec-15 inhibition enhances anti-tumor immune responses within the TME [7]. In our cohort of gastric cancer patients, cytoplasmic and/or membranous Siglec-15 expression was observed in 50.7% of tumor samples, and 36.4% of cases exhibited Siglec-15 positivity in TAMs. To our knowledge, this is the first study to evaluate Siglec-15 expression in gastric cancer within the Jordanian population. Our results are consistent with those of Quirino et al [10], who reported Siglec-15 expression in 61.9% of gastric cancer tumors and 42.5% of TAMs. Our results are further supported by transcriptomic data indicating elevated Siglec-15 mRNA levels in gastric cancer [9].

The overexpression of Siglec-15 has been identified in a range of malignancies, including bladder, breast, thyroid, esophageal, renal, hepatocellular, and lung cancers [6, 9]. For instance, Yang et al [13] found Siglec-15 expression on both tumor cells and TAMs in clear cell renal cell carcinoma. Additionally, Siglec-15 expression was present in both TAMs (13.3%) and neoplastic cells (22.8%) of lung cancer samples [7]. Similarly, Li et al [9] observed membranous Siglec-15 expression in 16.5% of lung adenocarcinoma cases. Shafi et al [11] further demonstrated significant Siglec-15 expression in TAMs and tumor cells across multiple cancers, including breast, lung, head and neck, and bladder cancers. Notably, TAM expression consistently exceeded tumor cell expression in those cancers [11]. In contrast, our findings revealed a higher frequency of Siglec-15 positivity in cancer cells than in TAMs. This discrepancy may stem from methodological differences, including the use of varying antibody types and different cut-off values.

Siglec-15 exerts immunosuppressive effects through mechanisms distinct from PD-1/PD-L1, offering potential therapeutic advantages for patients who do not respond to anti-PD-1/PD-L1 therapies or exhibit resistance [7]. Siglec-15 and PD-L1 typically showed mutually exclusive expression [7, 11], although Yang et al [13] found no association in clear cell renal cell carcinoma. In our gastric cancer cohort, no significant association was observed between Siglec-15 and PD-L1, likely due to the small sample size.

In this study, a trend toward poorer prognosis with positive Siglec-15 expression was observed, although the association did not reach statistical significance. Notably, 5-year survival rates were markedly lower in patients with cytoplasmic (36.3%) or membranous (37.6%) Siglec-15 expression, compared to the survival rate in patients with negative Siglec-15 expression (53.8% and 51.3%, respectively). Li et al [9] reported similar findings, linking Siglec-15 expression to unfavorable outcomes. In contrast, studies by Quirino et al [10] in gastric cancer and Hao et al [14] in lung cancer found a positive association between positive Siglec-15 expression and patient survival. These discrepancies underscore the need for further large-scale, multi-center investigations to clarify the prognostic significance of Siglec-15 across tumors.

Siglec-15 expression was not significantly associated with most clinicopathological features in our cohort. The only significant finding was an association between membranous Siglec-15 expression and tumor differentiation (P = 0.048), with higher frequency of positivity observed in moderately differentiated tumors. This is consistent with studies by Quirino et al, Shafi et al, and Yang et al [10, 11, 13]. While previous reports have linked Siglec-15 to factors such as lymphovascular invasion and TNM stage, findings remain inconsistent [10, 11, 15]. In our data, cytoplasmic expression showed a non-significant trend toward association with lymphovascular invasion (P = 0.075) and absence of metastasis (P = 0.068).

This study investigated Siglec-15 expression in gastric cancer patients to assess its potential as an immunotherapeutic target and to explore its association with clinicopathological features. Limitations of this study include the small sample size, reliance on a single method for detecting Siglec-15 expression, and the use of anti-Siglec-15 polyclonal antibodies without independent validation against an alternative detection method. Finally, the cohort was drawn from a single ethnic population, which may limit the generalizability of these findings to other ethnicities, particularly Asian populations in whom gastric cancer prevalence is considerably higher.

In conclusion, this study found elevated Siglec-15 protein expression in half of the patients, with a significant association only with histological grade. While Siglec-15 expression in cancer cells was linked to lower overall survival, this relationship was not statistically significant. Survival analysis showed lower survival rates in patients with older age (P = 0.0108), higher clinical stage (P = 0.0049), and higher pT stage (P = 0.0252). Additionally, cytoplasmic Siglec-15 expression showed trends toward associations with lymphovascular invasion (P = 0.075) and absence of metastasis (P = 0.068). Future studies with larger sample sizes are needed to better evaluate the relationship between Siglec-15 expression and clinicopathological features.

## Data Availability

The data supporting the findings of this study are available from the corresponding author upon reasonable request.
